# The Buffalo Medical and Surgical Library Association

**Published:** 1884-12

**Authors:** James W. Putnam

**Affiliations:** Secretary


### The Buffalo Medical and Surgical Library Association.

We direct the attention of our city readers to the circular calling together the members of the library association. The efforts of the profession should be actively enlisted in behalf of this enterprise, and we hope the active interest inspired from the coming meeting will encourage the directors to go on with their work in furnishing the latest literature on medical topics to the profession of this city.

The third annual meeting of the association will be held at the library, corner Pearl and Chippewa streets, on Friday evening, December 5th. Election of officers for the ensuing year, reports of Treasurer and Librarian will be presented, and other important business will be transacted. A large attendance is requested.

James W. Putnam, *Secretary.*

